# FOXM1: A novel drug target in gastroenteropancreatic neuroendocrine tumors

**DOI:** 10.18632/oncotarget.3600

**Published:** 2015-03-15

**Authors:** Franziska Briest, Erika Berg, Irina Grass, Helma Freitag, Daniel Kaemmerer, Florentine Lewens, Friederike Christen, Ruza Arsenic, Annelore Altendorf-Hofmann, Almut Kunze, Jörg Sänger, Thomas Knösel, Britta Siegmund, Michael Hummel, Patricia Grabowski

**Affiliations:** ^1^ Department of Gastroenterology, Infectious Diseases, Rheumatology CC13, Medizinische Klinik 1, CBF, Germany; ^2^ Institute of Pathology, CBF, Charité - Universitätsmedizin Berlin, Germany; ^3^ Department of Chemistry and Biochemistry, Freie Universität (FU) Berlin, Germany; ^4^ Institute of Pathology, Ludwig-Maximilians-Universität (LMU), Munich, Germany; ^5^ Department of General and Visceral Surgery, Zentralklinik Bad Berka GmbH, Germany; ^6^ Department of Internal Oncology and Hematology, Zentralklinik Bad Berka GmbH, Germany; ^7^ Institute of Pathology, Bad Berka, Germany; ^8^ Department of General, Visceral and Vascular Surgery, Friedrich-Schiller-Universität (FSU) Jena, Germany; ^9^ Institute of Pathology, CCM, Charité-Universitätsmedizin Berlin, Germany; ^10^ Institute of Biology, Humboldt-Universität Berlin, Germany

**Keywords:** gastroenteropancreatic neuroendocrine neoplasms, FOXM1, siomycin A, differentiation, cancer signaling

## Abstract

Gastroenteropancreatic neuroendocrine neoplasms (GEP-NENs) are heterogeneous tumors that need to be molecularly defined to obtain novel therapeutic options. Forkheadbox protein M1 (FOXM1) is a crucial transcription factor in neoplastic cells and has been associated with differentiation and proliferation. We found that FOXM1 is strongly associated with tumor differentiation and occurrence of metastases in gastrointestinal NENs. *In vitro* inhibition by the FOXM1 inhibitor siomycin A led to down-regulation of mitotic proteins and resulted in a strong inhibitory effect. Siomycin A decreased mitosis rate, induced apoptosis in GEP-NEN cell lines and exerts synergistic effects with chemotherapy. FOXM1 is associated with clinical outcome and FOXM1 inhibition impairs survival *in vitro.* We therefore propose FOXM1 as novel therapeutic target in GEP-NENs.

## INTRODUCTION

Gastroenteropancreatic neuroendocrine neoplasias (GEP-NENs) are heterogeneous tumors of the gastrointestinal system and the pancreas with limited therapeutic options, possibly due to crosstalks that re-activate mitogen signaling [1]. The search for novel “druggable” targets, therapeutic strategies and prognostic markers remains a considerable challenge.

Forkheadbox protein M1 (FOXM1) is regarded to be a crucial transcription factor in a plethora of solid cancers. As one of the early up-regulated proteins in cancerogenesis, FOXM1 has been demonstrated to contribute to all hallmarks of cancer [2-6]. It is considered a key regulator of the G2/M transition of the cell cycle and of the mitotic spindle integrity by regulation of cyclin A and B, cdc25B, aurora A and B kinases, survivin, PLK1, SKP2, CENPB and CENPF/A/B, and degradation of p21 and p27 [3, 4, 6-13]. FOXM1 also triggers cancer progression by promoting a VEGF-dependent angiogenic switch [14, 15] and by facilitating invasion via MMP-2 and MMP-9 secretion [16-18]. FOXM1 can be repressed by wild type p53 [19] and by FOXO3a [20-22]. It is then a further downstream effector of the PI3K-AKT-FOXO-axis, which is frequently deregulated in GEP-NENs [1, 23, 24]. It is also regulated by cell cycle proteins such as CDK4/6 [25]. The role of FOXM1 in neuroendocrine neoplasms has rarely been explored to date, but it has been recently described as marker for subtyping neuroendocrine lung cancer [26].

In this study we demonstrate that FOXM1 expression is associated with proliferation, differentiation and metastasis in gastrointestinal NEN and that inhibition of FOXM1 is a potential new therapeutic option.

## RESULTS

### FOXM1expression correlates with differentiation and metastasis in gastrointestinal NENs

**Figure 1:**
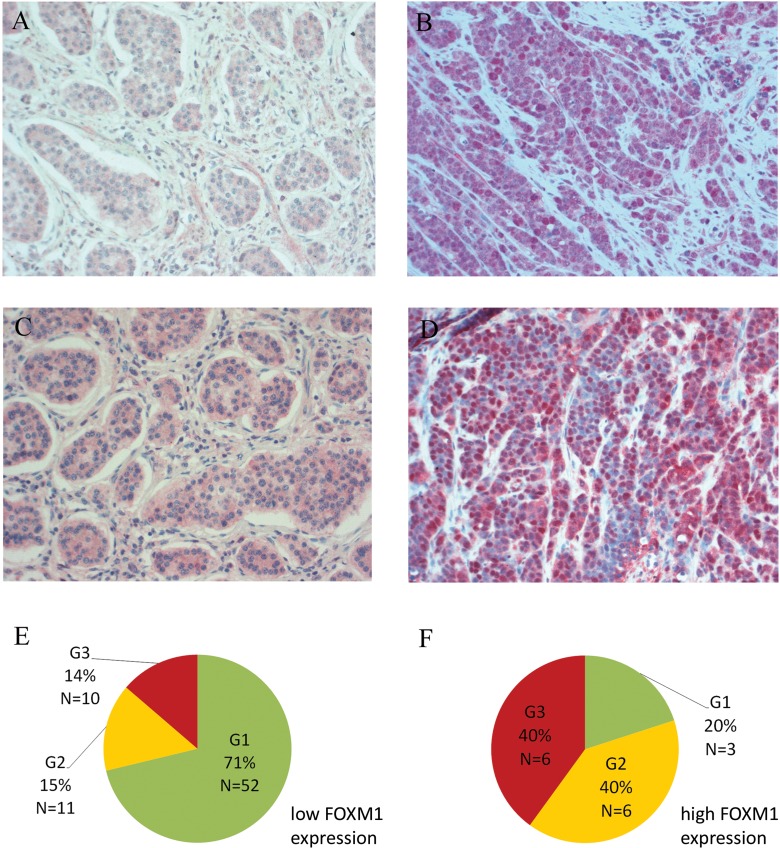
Immunohistochemical staining of FOXM1 **A.** Weak cytoplasmic and negative nuclear FoxM1 and **C.** negative nuclear STAT3 staining in a well differentiated ileal G1 NEN with liver metastasis. **B.** Strong FOXM1 (nuclear and cytoplasmic immunoreactivity) and **D.** nuclear STAT3 staining of a poorly differentiated gastric G3 NEC with liver metastasis. Nuclear localization of the oncogenic transcription factor STAT3 is associated with high FOXM1 expression (*p*=0.001). All pictures: light microscopy, 200x magnification. **E**+**F.** Distribution of WHO 2010 grading subgroups within the two FOXM1 expression groups of gastrointestinal NENs (N=88): in the FOXM1 low expression group, 71% of the tumors were graded G1, G2 tumors represented 15% and G3 tumors 14%. The FOXM1 high expression group is predominated by G2 and G3 tumors with 40% each. Only 20% of this group were G1 tumors.

First, we assessed the clinical relevancy of FOXM1 in GEP-NEN tumor specimens (summarized in Table 1). High FOXM1 staining (Figure 1) was detected in 30/131 (22.9%) of the GEP-NENs tissues. As only 3 specimens of the pancreatic subgroup were poorly differentiated, we focused on the analysis of the 88 specimens of gastrointestinal primary localization.

In this group, FOXM1 expression correlated with tumor differentiation according to WHO 2010 classification (refer to Table 2). In an univariate analysis, we found that 9/72 (12.5%) of the well-differentiated (G1 and G2) and 6/16 (37.5%) of the poorly-differentiated (G3) specimens had high FOXM1 expression (fisher's exact test: p=0.026). Interestingly, we found a strong difference of FOXM1 expression between G1 (5.5%) and G2 (35.3%) well-differentiated gastrointestinal NENs (fisher's exact test: *p*=0.004). The latter FOXM1 expression was similar to those tumors with G3 grading. Accordingly, gastrointestinal NENs can be subgrouped by a strong FOXM1 expression increase with the most significant cut off between 2% to 4% of the proliferation marker Ki-67 (*p*=0.000, Figure 1E and F).

In a subsequent univariate analysis, we could also demonstrate that FOXM1 expression is associated with the occurrence of metastasis in gastrointestinal NENs as 4/49 (8.2%) of the M0 subgroup and 11/36 (30.6%) of the M1 subgroup showed high FOXM1 expression (N=85; *p*=0.007).

**Table 1:** Summary of the most relevant results of the immunohistochemical analyses: number of analyzed specimens is indicated in the table: 88 gastrointestinal NEN specimens were stained for FOXM1, 49 and 36 pancreatic and gastrointestinal cases were analyzed for survivin and STAT3, respectively Lower numbers in the metastatic status analyses resulted from 3 cases with unknown status. All markers could be associated with metastatic status, and FOXM1 and nuclear survivin could be significantly linked to differentiation. FOXM1 further significantly correlated with survivin and STAT3 high nuclear staining.

Parameters	FOXM1 expression	Nuclear STAT3	Nuclear survivin	Differentiation	Metastatic status
FOXM1 expression		N=36 *p*=0.001*	N=49 *p*=0.030*	N=88 *p*=0.026*°	N=85 *p*=0.007*°
Nuclear STAT3	° assessed only in gastrointestinal NENs * univarite analysis		N.D.	N=36 not sig.*	N=36 *p*=0.007* p=0.011**
Nuclear survivin	**multivariate analysis			N=49 *p*=0.000*	N=46 *p*=0.000*

**Table 2:** Clinicopathological data of immunohistochemically analyzed gastrointestinal GEP-NENs: Distribution of localization, grading, differentiation and metastatic status More detailed information about clinicopathological data of all 131 patients can be found in Supplement 3.

Grading (WHO 2010)		G1 Ki-67 ≤ 2	G2 Ki-67 = 3-20	G3 Ki-67 > 20	total
primary	Ileum	38	12	1	51
	Colon	9	2	8	19
	Rectum	4	1	6	11
	other	4	2	1	7
differentiation	well	55	17	0	72
	poorly	0	0	16	16
metastatic status	M0	38	6	5	49
	M1	14	11	11	36
	N/A	3	0	0	3
total		55	17	16	88

### FOXM1 is up-regulated jointly with STAT3 and survivin in GEP-NEN

In order to determine how FOXM1 is co-regulated with other oncogenes, we chose (potential) upstream and downstream mediator of FOXM1. Immunohistochemical analysis was used to determine the expression status of STAT3 in 36 and of survivin in 49 cases of pancreatic and gastrointestinal NENs, respectively. The high FOXM1 expression could be linked to a high survivin nuclear localization (fisher's test: *p*=0.030; for gastrointestinal NENs only: *p*=0,029) which was up-regulated in 16/49 (32.7%) cases [27] of the study and was associated with both, differentiation and metastatic status (both: *p*=0.000).

High FOXM1 expression could further be linked to STAT3 nuclear localization (Figure 1D) in a fisher's exact test (*p*=0.001; for gastrointestinal NENs only: *p*=0.005). Nuclear STAT3 furthermore correlated with metastatic status in NENs (*p*=0,007; for gastrointestinal NENs only: *p*=0.034). In a multivariate analysis we analyzed the dependency of metastatic status from STAT3 and FOXM1 expression. In a binary logistic regression, STAT3 was determined to be an independent parameter with an odds ratio of 6.72 (*p*=0.011) in forward and backward stepwise regression, indicating that FOXM1 may be under transcriptional control of STAT3.

A correlation between FOXM1 and cytoplasmic survivin (p=1.00) or cytoplasmic STAT3 (*p*>0.428) and with phospho STAT3 (*p*>0.072) expression were not significant in our analyses (data not shown).

**Figure 2:**
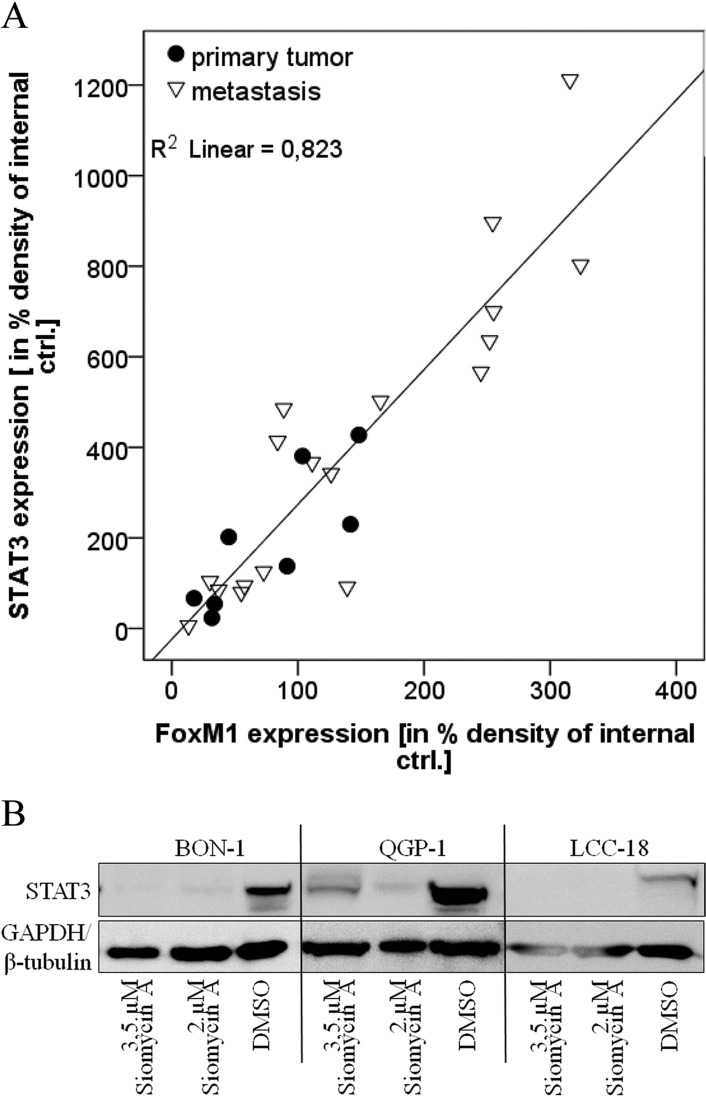
Role of STAT3 expression **A.** Primary tumor samples were lysed, analyzed by western blot in three independent experiments and densitometrically assessed. Statistical evaluation was done by linear multivariable regression and by non-parametric Mann-Whitney U Test, respectively. On tissue level, FOXM1 expression strongly correlated with STAT3 expression (*R square*= 0.823, *adjusted R square*=0.816) by linear regression. STAT3 is therefore associated with FOXM1 expression. A tendency of both proteins to be expressed to a higher extent in metastasis, compared to primary tumor material can be assumed, but requires a larger number of tumor samples. **B.** BON, QGP-1 and LCC-18 cell lines were treated with 2 and 3.5μM siomycin A for 72h. Cells were lysed and lysates were prepared by western blot. Immunodetection of STAT3 showed a strong decrease of STAT3 expression after siomycin A treatment in all cell lines.

The strong correlation of FOXM1 and STAT3 expression could be confirmed in lysates obtained from frozen primary and metastasis tumor material of GEP-NEN patients. Here, FOXM1 was significantly associated with STAT3 expression (*p*=0.000; Figure 2A). We could further detect a tendency of FOXM1 and STAT3 to be higher expressed in the metastasis group of tumor tissue than in the primary tumor group (Figure 2). We therefore show that STAT3 and FOXM1 in GEP-NENs may have prognostic significance.

### Siomycin A treatment induces regulation of proteins that are involved in GEP-NEN tumor biology

**Figure 3:**
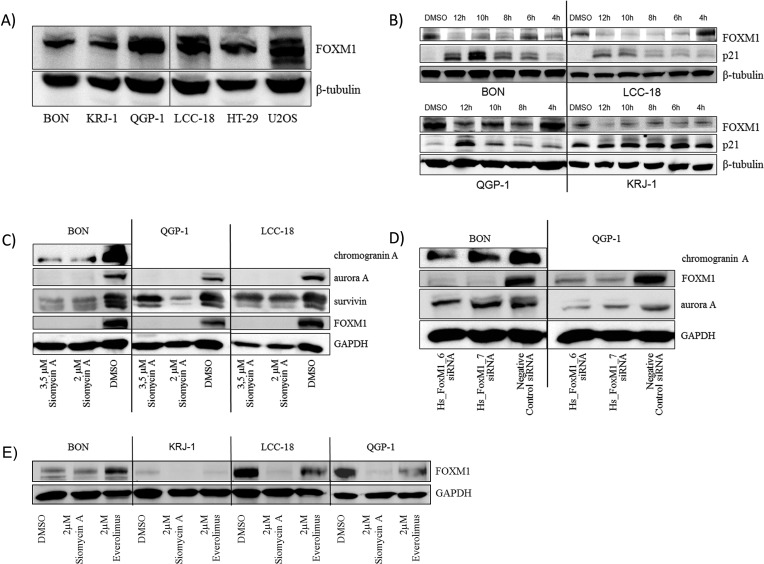
Western blot analysis of FOXM1 and potential target expression in GEP-NEN cell lines All cell lines including the non-neuroendocrine HT-29 and the FOXM1 overexpressing U2OS control cell lines expressed FOXM1 **A.** KRJ-1 cells, which is the only wild type *TP53* GEP-NEN cell line in this study (unpublished data) expressed the lowest level of FOXM1. After short time (12h) treatment with 10μM siomycin A, an increase of p21 expression was detected in a time-dependent manner **B.** Treatment of synchronized GEP-NEN cell lines with 2 and 3.5μM siomycin A for 72 hours resulted in a decrease of FOXM1, chromogranin A, aurora A and survivin expression **C.** Dependency of chromogranin A and aurora A down-regulation from FOXM1 depletion could be verifed by RNA interference with two different siRNAs targeting FOXM1 mRNA **D.** in BON and QGP-1 cells. Treatment with 2μM everolimus for 72 hours did not remarkably reduce FOXM1 expression **E**.

In our preliminary experiments, we have demonstrated that FOXM1 is expressed in GEP-NENs to a high extent and can be correlated to differentiation and the occurrence of metastasis in gastrointestinal NEN. Until now, effective therapy options for these tumors are not available. We therefore analyzed the effect of proteasome inhibitors, which target FOXM1 [28-31], *in vitro*. To initiate our studies, we assessed the basal expression of FOXM1 in the GEP NEN cell lines BON, QGP-1, KRJ-1, LCC-18 by western blot. All cell lines expressed detectable levels of FOXM1 (Figure 3A).

We chose the natural thiazole antibiotic, siomycin A and evaluated its effect on FOXM1 expression in treated GEP-NEN cell lines. We could demonstrate that FOXM1 was down-regulated time-dependently in all cell lines and that the cell cycle regulator p21 was up-regulated simultaneously (Figure 3B). Siomycin A is thus competent to inhibit FOXM1 in GEP-NEN cell lines and might influence the cell cycle regulation of GEP-NEN cells.

Chromogranin A is a common clinical neuroendocrine marker. Aurora kinases and survivin are mitosis associated proteins, the latter with a strong prognostic potential in GEP-NENs. Through western blot analyses, we found that chromogranin A, survivin, and aurora A were synchronously down-regulated after siomycin A treatment (Figure 3C). FOXM1 dependent down-regulation of aurora A and chromogranin A could be further confirmed by determining the expression after knockdown of FOXM1 by RNA interference (Figure 3D). Everolimus did not exert mentionable effects on FOXM1 expression (Figure 3E), as it affects the mTOR signaling and is not considered to be involved in FOXM1 regulation.

Interestingly, we found STAT3 also down-regulated under siomycin A treatment, which reveals some insight into the mode of action of this natural agent (Figure 2B).

### Siomycin A treatment induces antiproliferative effects on GEP-NEN cell lines *in vitro*

To assess siomycin A not only as modulator of neuroendocrine markers and proliferation regulators, but also as effective treatment option for GEP-NENs, we calculated the dose of half maximal inhibitory effect (IC50) of siomycin A and everolimus for each GEP-NEN cell line (Suppl. 1). Everolimus was chosen because it is one of only 3 molecular therapy options that have been approved for GEP-NEN treatment. The IC50 of siomycin A was determined as ~1μM for BON and LCC-18 cells and ~2μM for QGP-1 cells and are consistent with the already published IC50 values for this natural agent in other cells lines *in vitro* [32]. We could not determine an IC50 for KRJ-1 cells due to the interference of native cellular clustering of this non-adherent cell line. We hypothesize that the surface cell layer of the spherical cell clusters protected the inner cells from the treatment and gave an incalculable growth advantage increasing with the size of the clusters. For these cells we estimated an IC50 similar to those of the other GEP NEN cell lines.

**Figure 4:**
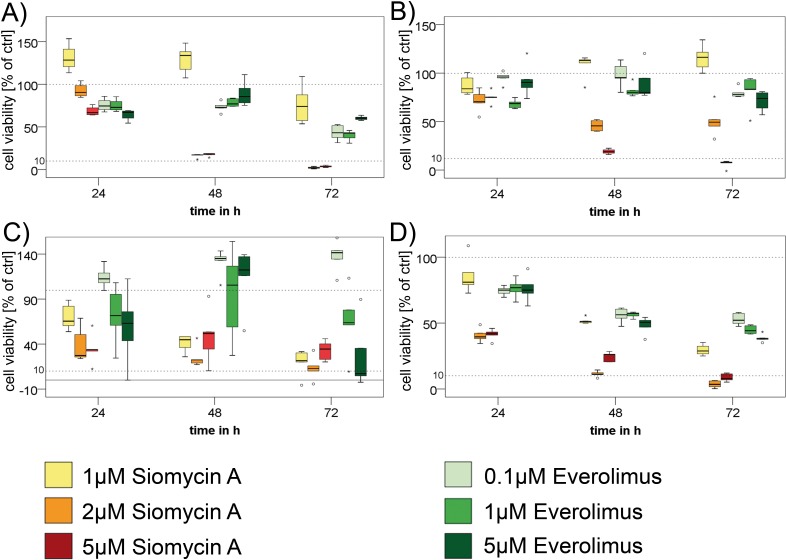
Treatment of GEP-NEN cell lines with siomycin A In three independent experiments, BON **A.**, QGP-1 **B.**, KRJ-1 **C.** and LCC-18 **D.** cells were treated with increasing concentrations (1μM, 2μM, 5μM) of siomycin A and (0.1μM,1μM, 5μM) everolimus for 24, 48 and 72 hours and analyzed by colorimetric proliferation assays (graphs in percent of internal DMSO controls). Siomycin A significantly inhibits GEP-NEN cell proliferation (*p=*0.000) and was superior to everolimus treatment in all cell lines. Antiproliferative effects could be shown for concentration ≥1μM in BON, KRJ 1, LCC 18 and ≥2μM for QGP-1 cells in relation to DMSO internal controls and were verified in a linear regression analysis. KRJ-1 cells showed a strong variance in the response due to intercellular clustering effects. Nevertheless these changes were highly significant (*p*=0.000). Representative data of one experiment is shown. Dotted lines indicate 100% and 10% of control.

We could demonstrate a significant antiproliferative effect of siomycin A on GEP-NEN cell lines *p*=0.000; Figure 4). Siomycin A is therefore superior even to equal doses of everolimus, which only showed significant impact on the cell lines BON (*p*=0.008) and LCC-18 (*p*=0.000), whereas QGP-1 (*p*=0.092) and KRJ-1 (*p*= 0.38) did not significantly respond to everolimus even in concentrations equal to siomycin A treatment (Figure 4).

### Siomycin A reduces mitosis and induces apoptosis in GEP-NEN cell lines *in vitro*

**Figure 5:**
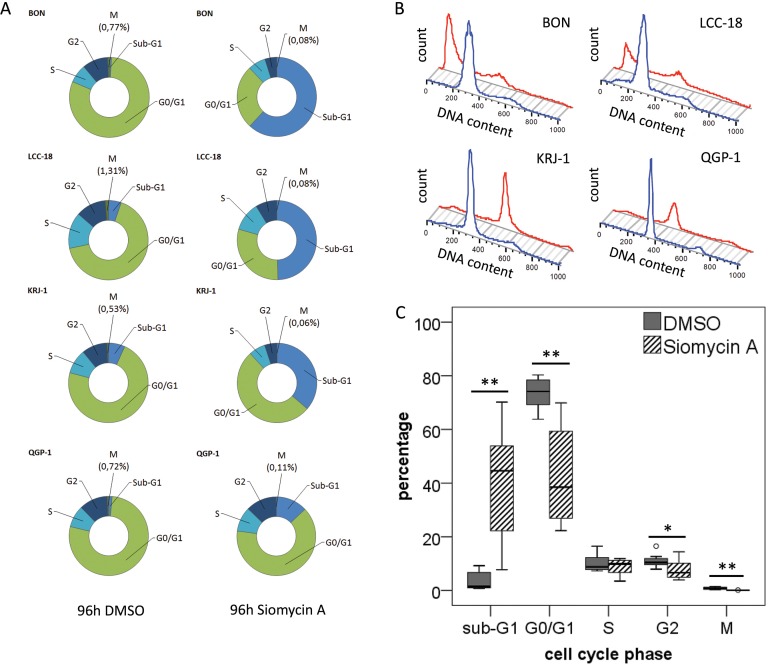
Cell cycle analysis of GEP-NEN cell lines treated with siomycin A Unsynchronized BON, QGP-1, KRJ-1, LCC-18 cells were treated with 2μM siomycin A for 96h and analyzed by mitotic index flow cytometry. Cells were stained with an anti-phospho-H3 antibody and propidium iodide and analyzed versus 0.04% DMSO controls. All changes were calculated with respect to DMSO controls. **A.** Average of cell cycle changes in the distinct GEP-NEN cell lines after Siomycin A treatment. BON cells show a strong 72-fold increase in the sub-G1 cell death population; in KRJ-1, QGP-1 and LCC-18 cells a 5- to 9-fold increase could be detected. Most notably, the number of mitotic cells (indicated by phosphorylated histone H3) significantly decreases after siomycin A treatment in the range of 6.5- to 16-fold. **B.** Representative histogram of one 96h incubation experiment, blue line indicates the DMSO control, red lines visualizes the cell cycle histogram after siomycin A treatment (more detailed flow cytometry data can be found in Suppl. 2). The average chances of all cell lines under siomycin A treatment are visualized in **C.**, all significant values are indicated by stars. Double starred bars indicate significance levels *P*≤1%.

As FOXM1 is strongly associated with mitotic regulation, we wanted to understand the impact of siomycin A on the cell cycle of GEP-NEN cells. After 96h of treatment, the majority of BON, and LCC-18 and a large population of KRJ-1 cells analyzed by mitotic index flow cytometry, have undergone apoptosis or necrosis (indicated as Sub-G1) and showed a significantly decreased mitotic population (*p*<0.001; Figure 5). QGP-1 cells only showed a moderate increase in apoptotic or necrotic cells and an average 6-fold decrease of the mitotic cell population could be detected (Figure 5 and Suppl. 2).

**Figure 6:**
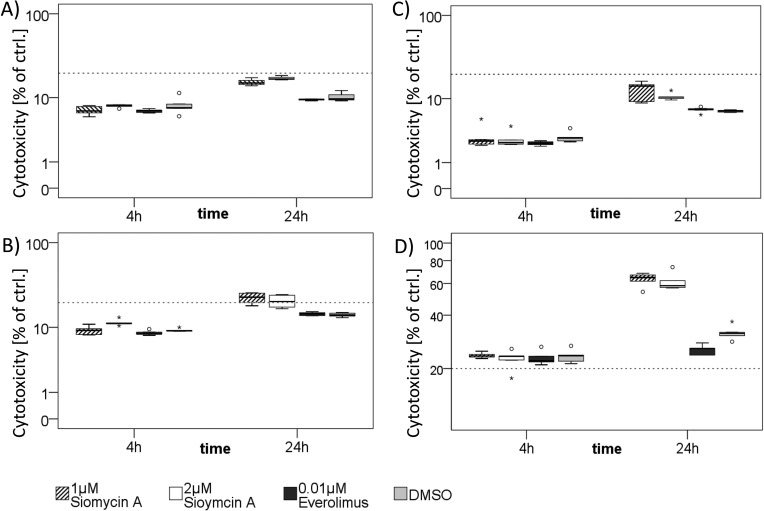
Cytotoxic effects of siomycin A treatment *in vitro* BON **A.**, LCC-18 **B.**, QGP-1 **C.**, and KRJ-1 **D.** cells were treated with 1 and 2μM siomycin A versus 0.04% DMSO, 0.01μM Everolimus and 1% Triton X (total cell lysis control) for 4 and 24 hours. LDH release from necrotic cells was colorimetrically measured in % of Triton X lysed cell control by LDH cytotoxicity assay. Dotted line indicates 20%. BON and LCC-18 cells showed very low spontaneous cytotoxicity (lower than 20%). QGP-1 cells had cytotoxic effects in the range of 20%. Only KRJ-1 cells had higher cytotoxicity values up to 63%. Further studies should be performed to assess the *in vivo* tolerability of siomycin A.

To distinguish whether cells undergo apoptosis or necrosis, we completed our analyses by an LDH-based cytotoxicity assay. Siomycin A in effective doses showed a low cytotoxic effect on BON, QGP-1 and LCC-18 (Figure 6 A-C). Only in KRJ-1 there were strong cytotoxic effects, presumably due to the loss of intercellular contact. As resuspending the cell clusters was obligatory for equal cell numbers and the time of incubation was too short for a reattachment, induction of anoikis might be an explanation for an early and strong loss of membrane integrity (Figure 6D).

We conclude that siomycin A predominantly induces a decrease of mitotic activity and apoptosis in GEP-NEN cell lines and its *in vivo* tolerability should be further assessed in animal studies.

### Siomycin A induces synergistic effects combined with chemotherapy

**Figure 7:**
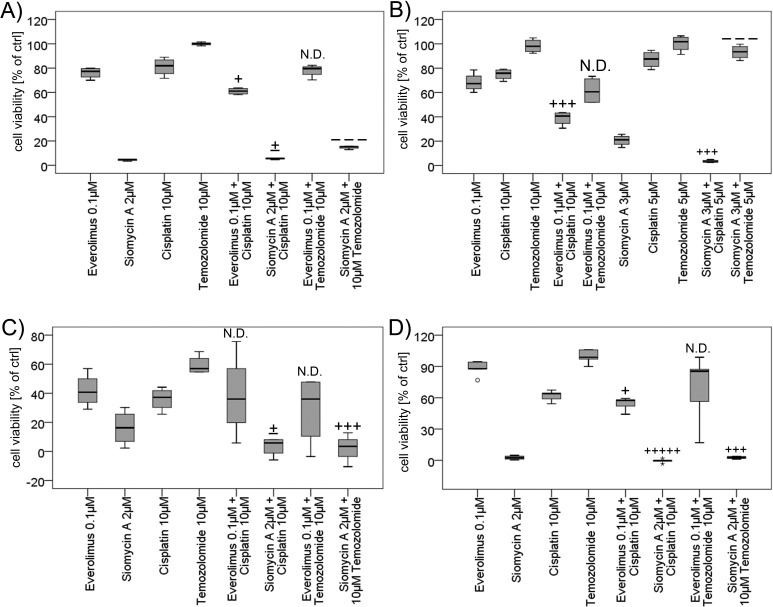
Combined treatment of GEP-NEN cell lines with siomycin or everolimus and genotoxic drugs BON **A.** KRJ-1 **C.** and LCC-18 **D.** cells were treated with 2μM siomycin A or 0.1μM everolimus alone and combined with 10μM cisplatin or temozolomide for 72 h versus 0.1% DMSO. QGP-1 cells **B.** were treated with 3μM siomycin A combined to 5μM of cisplatin or temozolomide. Proliferation was analyzed by colorimetric WST proliferation assays (graphs in percent of internal DMSO controls) and mean combination index was calculated using the Chou and Talalay method by CompuSyn 1.0 software [35, 36]. Everolimus combined to cisplatin showed *slight synergism* to *synergism* (BON: CI=0.864; QGP-1: CI=0.457; KRJ-1: n.d.; LCC 18: CI=0.862) after 72 hours of treatment. Siomycin A combined to cisplatin induced *nearly additive effects to very strong synergisms* in GEP-NEN cell lines (BON: CI=0.996; QGP-1: CI=0.548; KRJ-1: CI=1.066; LCC-18: CI=0.062) and the combination with temozolomide was *antagonistic* in pancreatic BON (CI=1.538) and QGP-1 cells (CI=2.627), and *synergistic* in gastrointestinal KRJ-1 (CI=0.526) and LCC-18 (CI=0.645) cell lines. (Captions [35]: very *strong synergism*: +++++; *strong synergism*: ++++; *synergism*: +++; *moderate synergism*: ++; *slight synergism*: +; *nearly additive*: +/−; *slight antagonism*: -, *moderate antagonism*: --; *antagonism*: ---; *strong antagonism*: ----; *very strong antagonism*: -----).

Siomycin A might not be used in monotherapy regimens, but inhibition of FOXM1 has been already assessed to have synergistic effects combined with genotoxic drugs [19, 33, 34]. We therefore examined the effect of siomycin A combined with cisplatin or temozolomide versus everolimus combined with chemotherapy. 10μM cisplatin induced moderate inhibitory effects in WST proliferation studies. 10μM Temozolomide did not inhibit cellular proliferation in BON, QGP-1 and LCC-18 cells and showed a moderate antiproliferative effect in KRJ-1 cells. Quantitated by the combination index method after Chou and Talalay [35, 36], we found *slight synergistic* to *synergistic* effects in all cell lines for 0.1μM everolimus combined with 10μM cisplatin after 72 hours of treatment (Figure 7). This favorable combination has been described before [37] and could be reproduced for GEP-NENs in this study. Nevertheless, even the combined everolimus treatment was less effective than the siomycin A monotherapy in all cell lines. Everolimus combined to temozolomide did not show enhanced effects.

2 or 3 μM Siomycin A combined to 10 or 5μM cisplatin, respectively, induced *nearly additive* to *very strong synergistic* inhibitory effects in GEP-NEN cell lines. Interestingly, the effect of siomycin A combined to temozolomide was *antagonistic* in pancreatic BON (Figure 7A) and QGP-1 cells (Figure 7B), whereas the gastrointestinal cell lines KRJ-1 (Figure 7C) and LCC-18 (Figure 7D) responded with *synergistically* reduced proliferation. Siomycin A combined to everolimus induced *antagonistic* effects and increased cellular proliferation in relation to DMSO controls (data not shown).

## DISCUSSION

GEP-NENs, in particular tumors that originate in the gut, lack tailored molecular therapies and biomarkers. Interestingly, the expression of several proteins, such as survivin, aurora kinases, p16(INK4A) and IGF-1, have been found altered in GEP-NENs, and are associated with FOXM1 expression in other cancer entities [38-40]. *FOXM1* has further been described as a crucial proto-oncogene. There are currently few prognostic markers and therapeutic options, especially in the NENs of the gut, and prognosis is only associated with the proliferation index indicated by Ki-67. We therefore assessed FOXM1 as a potential disease progression marker and therapy target.

We found FOXM1 significantly up-regulated in poorly differentiated tumors (*p*=0.026). FOXM1 could not be validated as a significant independent marker. This result may be due to its crucial role in mitosis and proliferation, which would influence the expression of proliferation markers, such as Ki-67.

Interestingly, the correlation of FOXM1 expression and Ki-67 staining reveals a low expression in the G1-graded tumors and FOXM1 expression significantly increases in the tumor subgroup with a Ki-67 value higher than 2-4% (*p*=0.000). There is currently an unclear Ki-67 cut-off value to distinguish G1 and G2 GEP-NENs, since former WHO classifications stated Ki-67 cut-off values from 2 to 5%, which were dependent from the localization of the primary tumor (WHO2000/2004). As Ki-67 is only a descriptive marker, these changes in the FOXM1 transcription factor expression might give a mechanistic explanation for the distinct clinical prognoses of both subgroups. Therefore, FOXM1 might serve as a secondary refining marker to discriminate between G1 and G2 gastrointestinal NENs.

FOXM1 expression could also be related to the occurrence of metastases (*p*=0.007) and showed a tendency to be up-regulated in primary metastases material. Therefore, we could show that FOXM1 is a progression associated protein in GEP-NENs. These data are consistent with already published results for FOXM1 as oncogene in many other cancer entities [41-43].

FOXM1 is described as a transcriptional target of STAT3 [44]. Here, we demonstrate that FOXM1 is up-regulated jointly with nuclear STAT3 in GEP-NENs. Although being localized in the nucleus, stained STAT-3 molecules were not consistently phosphorylated, as the parallel immunohistochemical staining with the phospho-STAT3 antibody was highly variable (data not shown). We hypothesize that STAT3 might transactivate *FOXM1* expression in GEP-NENs. These results may be reflected in recent studies showing that unphosphorylated STAT3 (U-STAT3) can be shuttled into the nucleus by importin-alpha3 and -alpha6 and is crucially involved in cancer signal transduction [45]. It has been demonstrated to cooperate with other transcription factors such as unphosphorylated NF-kappaB to bind to DNA and transactivate target genes [46, 47]. Furthermore, U-STAT3 can mediate FOXO3a nuclear export and thus FOXO3a inactivation and FOXM1 activation, whereas phosphorylated STAT3 re-localizes FOXO3a into the nucleus and therefore promotes its FOXM1 antagonistic activity [48].

Thiazole compounds, such as siomycin A, have been assessed as promising FOXM1 inhibitors with little impact on untransformed cells [49]. In general, proteasome inhibitors might stabilize a hypothetical negative regulator of FOXM1 [29, 32, 50]. In this study, siomycin A treatment decreases the expression of both, STAT3 and FOXM1, although the mechanism of action is relatively unknown [4, 29, 30]. Therefore it is possible that the proteasome inhibitor sioymcin A targets FOXM1 indirectly by a JNK-STAT3-dependent mechanism [31, 51]. This may explain the effectiveness of siomycin A, as STAT3 has been shown to interfere with FOXO proteins [48]. Thus, siomycin A might interfere with STAT3, which contributes to FOXO3a nuclear localization and results in *FOXM1* repression and inhibition of mitosis.

In our study we have further confirmed that survivin and aurora kinases are FOXM1 targets. This is not a novel result, but as aurora kinases have previously been described as druggable targets in GEP-NENs [52, 53], novel combinatory treatments are conceivable. Furthermore, the fact that aurora A kinase and survivin are down-regulated under FOXM1 inhibition supports the notion that the mitotic instability, and not the (in-)activation of various kinases, may be the better approach for the treatment of GEP-NENs.

Independently from its impact on neuroendocrine tumor signaling, we could show the natural agent siomycin A as effective therapeutic option when applied in the range of 1-2μM. We hypothesize that the higher IC50 values in QGP-1 are related to a *TP53* frameshift mutation and thus loss of p53 expression (unpublished data) which impairs apoptosis induction. In all cell lines, siomycin A shows a strong maximum effect, but presumably due to its pharmacochemical characteristics, it must be applied in relatively high doses *in vitro* [32]. Notably, is has been described not to affect normal cells [29, 49, 54, 55]. We could demonstrate that siomycin A treatment reduces mitosis and induces low unspecific cell cytotoxicity in three cell lines. We further showed beneficial combinatory effects combined to cisplatin in all cell lines and combined with temozolomide in the gastrointestinal cells. As FOXM1 expression is critically linked to DNA damage signaling and p53 status [33, 56], subtype specific prediction markers for combined therapy approaches should be further evaluated.

Therefore, the inhibition of FOXM1 by proteasome inhibitors is a potential therapeutic option in GEP-NENs which should be further evaluated.

In conclusion, we could demonstrate an association of FOXM1 with the proliferation index indicated by Ki-67 and with mitotic proteins that have been assessed as crucial players in GEP-NEN biology [27, 52, 53]. This effect could be confirmed by an overall decreased mitotic activity in cells after siomycin A treatment. We have further linked FOXM1 to STAT3 expression and metastasis.

Finally, we have demonstrated that FOXM1 inhibition by siomycin A showed a stronger effect on the tested GEP-NEN cell lines than everolimus. Given the fact that everolimus is a well-established therapy in pancreatic NENs, but its efficacy is limited, we propose a potential for the assessment of a combination therapy with FOXM1 inhibitors. FOXM1 inhibition should be also considered as a (combinatory) therapeutic approach especially in gastrointestinal NENs, where effective therapeutic options are currently not available. In this context, our data further provide a strong rationale for assessing other proteasome inhibitors, such as casticin, thiostrepton and bortezomib [36, 37] as therapeutic strategies.

In conclusion, FOXM1 may serve as a clinical prognostic factor and a therapeutic target for GEP-NENs.

## MATERIALS AND METHODS

### Patients and samples

For immunohistochemical analysis, 131 paraffin embedded specimens of pancreatic (n=43), ileal (n=51), colorectal (n=30) and gastric (n=7) neuroendocrine neoplasms have been retrospectively analyzed for FOXM1 expression. Cases (age 17-87 years) were collected and prepared as tissue microarray (TMA) at Universitätsklinikum Jena (n=82) and as whole section (3-5μm) tissue slides at Charité-Universitätsmedizin Berlin (n=49), respectively, with permission of the local ethical committees. The TMA was assembled using 0.6 cm punch biopsies from all samples according to standard procedures [57]. Five-year follow-up was complete in all 131 cases. Inclusion criteria for this study were: positive staining for neuroendocrine markers and availability of clinicopathological information. Tumors were re-classified and Ki-67 re-stained according to the WHO 2010 classification: 85 patients had G1 neuroendocrine neoplasms, 27 patients suffered from G2 NEN. 19 patients were diagnosed to have a G3 neuroendocrine cancer. Further clinicopathological information can be found in table 2 and supplement 3.

In addition, 36 specimens of the whole section cohort were selected for further staining against STAT3 and phospho-STAT3. Nuclear survivin (N=49) has been stained in the same cohort before [27].

In a further prospective study, 44 tissue samples of fresh frozen material of GEP-NENs derived from 15 patients were collected at the Department of General and Visceral Surgery at the Zentralklinik Bad Berka GmbH between 2009 and 2013 with an institutional review board approval for guidelines and ethical procedures. The diagnosis of NEN was based on immunohistochemical characterization of chromogranin A and synaptophysin expression, on proliferation index (Ki-67) and on morphological criteria according to the WHO 2010 grading classification. On-site fresh frozen tissue specimens were H&E (hematoxylin and eosin) stained and analyzed for tumor cell ratio and morphological features such as high immune cell invasion or vascularization. Only samples with tumor cell content higher than 80 percent were included. The samples were mechanically homogenized and lysed in NP-40 buffer.

Data derived from different samples of the same tumor were aggregated and mean values were calculated. Total number of analyzed tumors thus results in 26. Further clinicopathological data is provided in supplement 4.

### Cell lines

The following GEP-NEN cell lines were used for *in vitro* experiments: pancreatic: BON [58] and QGP-1 [59]; obtained from *Japanese Collection of Research Bioresources*), ileal: KRJ-1 [60] and colonic: LCC-18 [61].

All cell lines were authenticated (if indicated as unique) by genetic STR typing by the DSMZ, Braunschweig, Germany in 2012 and 2013 and only cells passaged not longer than 15 passages after receipt were used. Cells were tested periodically for maintained cell line specific expression of neuroendocrine markers (chromogranin A, synaptophysin, cytokeratin, vimentin, syntaxin) by immunofluorescence microscopy. All cell lines were grown in Quantum 263 for tumor cells (GE Healthcare Munich, Germany) including 1% penicillin/streptomycin or in cell line specific medium (containing 10% FBS gold and 1% penicillin/streptomycin): BON and KRJ-1 in Dulbecco's Modified Eagle Medium: Nutrient Mixture 1:1 F-12 (DMEM/F12) with stable glutamine, QGP-1 were cultured in RPMI 1640 with stable glutamine and LCC-18 were grown in DMEM 4.5g/l glucose with stable glutamine. The non-neuroendocrine HT-29 and the FOXM1 overexpressing U2OS cell lines were used as controls, if indicated.

### Western blot

SDS page and western blot of NP-40 lysed material was performed using a standard protocol and documented by ponceau S staining. Primary antibodies (obtained from Cell Signaling Technology Inc. Danvers, MA, USA: pan-actin (D18C11), phospho-Tyr705-STAT3; Santa Cruz Biotechnology Inc. Dallas, Texas, USA: FOXM1 (C-20), p21; GeneTex Inc. Irvine, CA, USA: GAPDH; Abcam plc Cambridge, UK: aurora A; PROGEN GmbH Heidelberg: chromogranin A (klon LK2H10)); BD Transduction Laboratories, Franklin Lakes, NJ, USA: STAT3; Sigma-Aldrich: beta-tubulin) and secondary antibodies (Dako Deutschland GmbH, Hamburg, Germany: swine anti rabbit IgG-HRP, goat anti-mouse IgG-HRP; Santa Cruz Biotechnology Inc: donkey anti-goat IgG-HRP) were applied. Antibody binding was detected with ECL™ *prime Western Blotting detection reagent* (Amersham™ GE healthcare) and documented by Fujifilm LAS-4000 luminescent image analyzer. For re-probing, membranes were incubated in acidic glycine buffer (0.2 M glycine, 1% SDS, 0.1% Tween 20, pH 2.2). Chemiluminescence signals were densitometrically detected with Multi Gauge V3.1. Values of three independent experiments were normalized to an internal control and statistical assessment was performed with IBM SPSS Statistics 20.

### Immunohistochemistry of paraffin-embedded specimens

Immunohistochemistry was performed by APAAP method. Antibody (FOXM1 C-20) was obtained from Santa Cruz Inc. and diluted to a working concentration of 2μg/ml. The scoring of FOXM1 was assessed by 1.) administration of a nuclear (based on a previously described method for FOXM1 nuclear staining in neuroendocrine lung cancer [26] and a cytoplasmic score; 2.) integrating both in one overall score. The cytoplasmic FOXM1 immunoreactivity was assessed as 0 (no staining), 1 (weak staining), 2 (moderate staining) and 3 (strong staining). Only moderate and high staining was included in the FOXM1 cytoplasmic positive score. Due to the fact that FOXM1 cytoplasmic staining is described as important parameter [62] and FOXM1 nuclear staining was presumed to be the more critical marker overall, thus we created a dichotomized score with a proper weighting of both parameters. Here, nuclear and cytoplasmic scores were summated and a score of “2” was defined as high staining. Consequently, both nuclear and cytoplasmic staining had to be 2 or greater to be assessed as “high FOXM1” expression. Immunohistochemical evaluation was done by two independent experts (P.G. and R.A.).

STAT3 antibody was obtained from BD biosciences and diluted to 1:100; phospho-(Tyr705)-STAT3 antibody was purchased from Cell Signaling Technology and administered in a 1:50 dilution. Score was assessed separately for nuclear and cytoplasmic staining as 0 (no), 1 (weak), 2 (moderate), 3 (strong) immunoreactivity. To dichotomize this variable, only moderate and high staining were included as STAT3 high expression. Scores and utilized antibodies for survivin have been previously described [27].

### Statistical analyses

Correlation analyses of continuous variables were analyzed by multivariate linear regression in forward and backward stepwise regression. For univariate analyses containing one or two dichotomized variables, the Mann-Whitney *U* test or the χ^2^ test were applied, respectively. For results with low expected counts due to low sample numbers, we applied the exact fisher's test, if possible. Multivariate analysis with dichotomized dependent variables was performed by binary logistic regression in forward and backward regression. Overall survival was estimated by the Kaplan-Meier method, starting from the time of diagnosis. The survival was evaluated by the Mantel-Cox log-rank test and in a multivariate analysis by Cox regression. Differences were considered to be significant for *p* < 0.05. All statistical analyzes were performed using IBM SPSS Statistics 20 software.

### GEP-NEN cell lines treatment with siomycin A vs. everolimus

For cell cycle synchronization, cells were incubated in 10μM thymidine (Sigma-Aldrich, St. Louis, MO, USA; in culture media) for 72 hours or starved 24 hours in culture medium containing 0.01% FBS.

Siomycin A was obtained from Sigma-Aldrich and dissolved in DMSO. Everolimus as control therapy was obtained from Cell Signaling Technology. For proliferation and cytotoxicity assays, 10000-20000 cells per well were used in quintuplets in 96 well plates and treated 4 and 24h for LDH and 24, 48 and 72h for WST-assay with different concentrations and controls. *WST-1 proliferation reagent* and *LDH cytotoxicity detection reagent* (Roche; Basel, Switzerland) were applied according to the manufactures' instructions. In the LDH assay, 3% FBS was used instead of 1%, as recommended in the instructions. Cell density was colorimetrically quantified with a multi-well spectrophotometer (TECAN sunrise™).

### *In vitro* RNAi knockdown of FOXM1

Cells were transfected with *Lipofectamine 2000* (Life technologies, Carlsbad, CA, USA) containing 40 pmol/ml siRNA (*FlexiTube siRNA* against human FOXM1 transcript NCBI Reference Sequence NM_001243088, NM_001243089, NM_021953, NM_202002, NM_202003; QIAGEN; Target Sequences: Hs_FOXM1_6: 5′-AACATCAGAGGAGGAACCTAA-3′,

Hs_FOXM1_7: 5′-TGGGATCAAGATTATT AACCA-3′) or *AllStars Negative Control* siRNA; both QIAGEN) according to the manufacturer's instructions for 72 h.

### Cell cycle analysis by mitotic index flow cytometry

Cells were seeded into 6-well plates and incubated unsynchronized with 2μM siomycin A for 96h. Cells fixed in 70% cold ethanol and washed in PBS containing 0,5% Tritin X and 1 % BSA. For intercellular antibody staining, cells were incubated for 1h in Phospho-(Serine-10)-Histone H3 antibody (Cell signaling #3377) diluted 1:6000 in PBS/Triton/BSA. Secondary antibody (Alexa Fluor goat anti-rabbit; Life technologies) was applied 1:500. Cells were incubated 30 min. in PBS containing 20μg/ml propidium iodide (Life technologies) and 10μg/ml RNase A. Flow cytometry was conducted with FACSCalibur (Becton Dickinson) by BD Cell Quest Pro software and analyzed with FlowJo 8.7 software.

### Combination therapy of GEP-NEN cell lines

Cells were grown, synchronized by reduced serum supply and seeded in quintuplets in 96 well plates. Cells were synchronously treated with increasing concentrations of Siomycin A, everolimus, cisplatin (Charité Berlin dispensary) or temozolomide (Santa Cruz Biotechnology Inc.) and 0.1% DSMO, respectively, to obtain dose response curves as recommended by the Chou-Talalay method [35]. Additionally, several non-constant combinations of >IC50 concentrations of siomycin A or everolimus and cisplatin or temozolomide were assessed. WST-1 proliferation assay (Roche; Basel, Switzerland) was performed colorimetrically quantified. Data was analyzed by CompuSyn 1.0 at Fa=0.5 [63] and IBM SPSS Statistics 22 software.

## SUPPLEMENTARY MATERIAL, FIGURES, TABLES


