# Supplementary material for: FOXM1: A novel drug target in gastroenteropancreatic neuroendocrine tumors
Source: Oncotarget. 2015 Mar 15;6(10):8185–99. doi: 10.18632/oncotarget.3600 (PMC4480744; doi:10.18632/oncotarget.3600)
Supplement: Supplementary file 1 [file oncotarget-06-8185-s001.pdf]

FOXM1: A novel drug target in gastroenteropancreatic neuroendocrine tumors

Supplementary Material

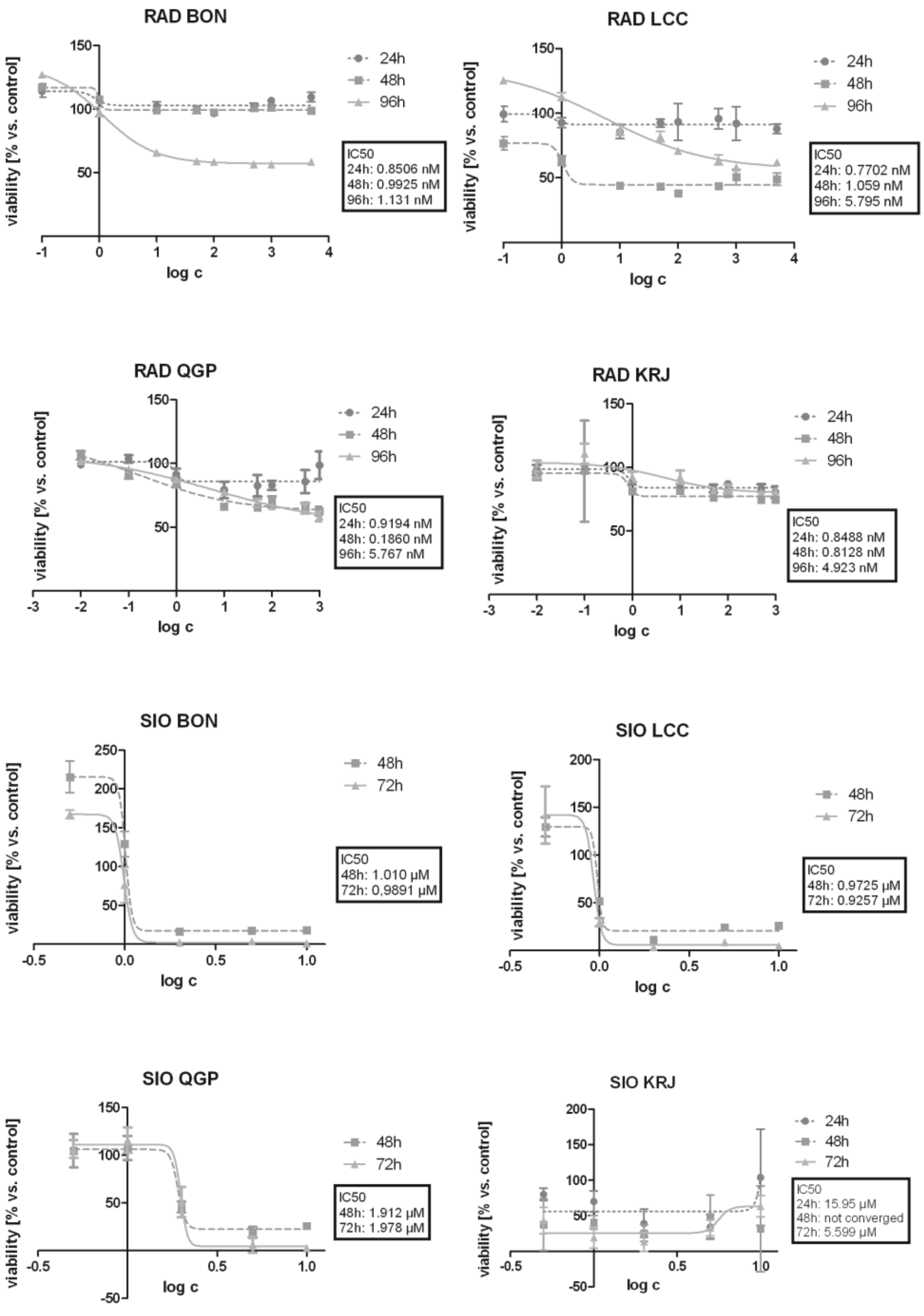

Suppl. 1: **Dose-response curves of BON, QGP-1, KRJ-1 and LCC-18 cells under everolimus (RAD001) and siomycin A treatment:** IC50 for everolimus was estimated as 1nM for all cell lines. IC50 for sioymcin A was estimated 1µM for BON-1, LCC-18 and KRJ-1 cells and 2µM for QGP-1 cells.

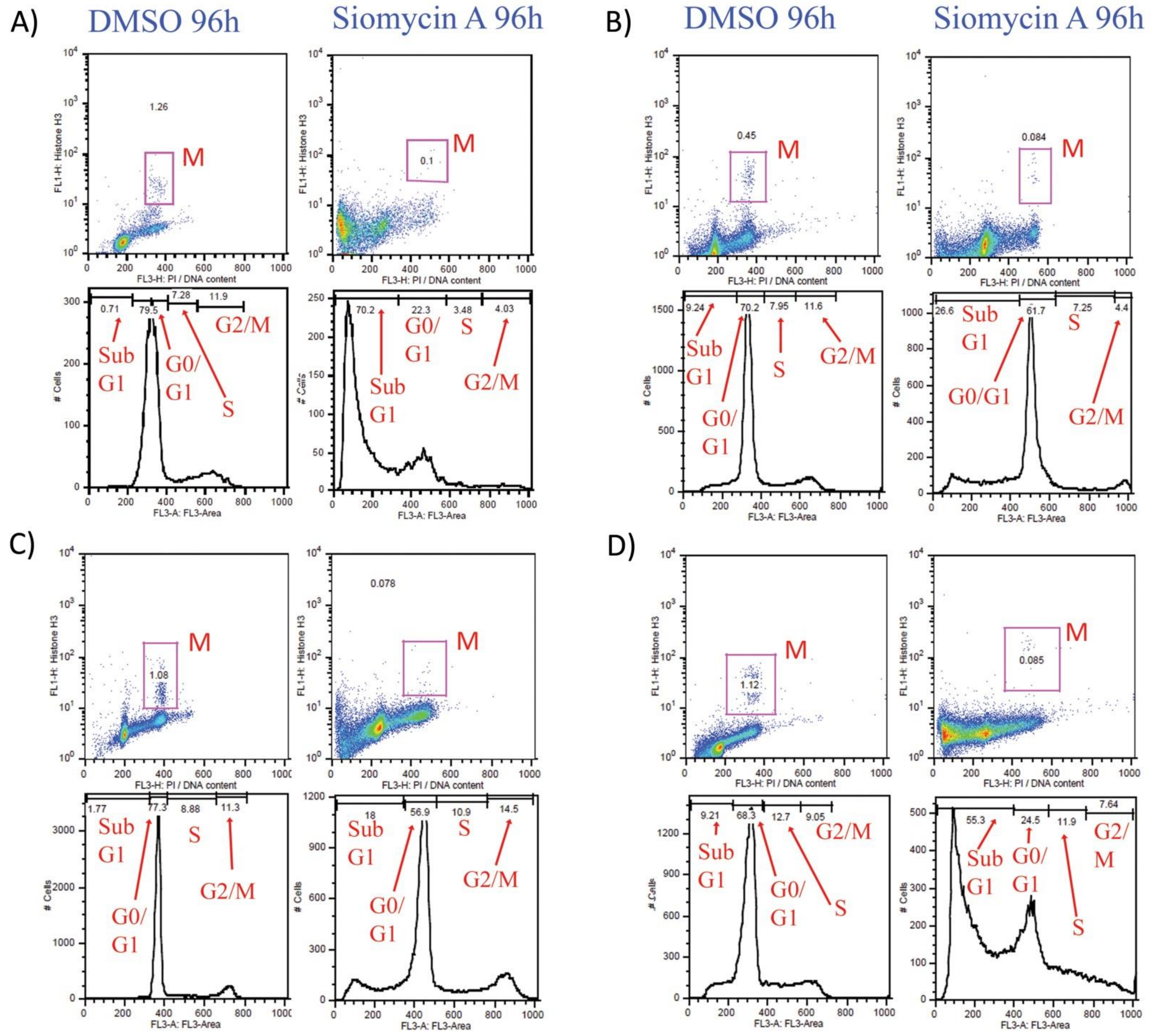

Suppl. 2: **Cell cycle analysis of GEP-NEN cell lines untreated and treated with siomycin A:** BON (A), KRJ-1 (B), QGP-1 (C), LCC-18 (D) were treated with siomycin A versus DMSO for 96h. Cells were fixed and stained with an anti-phospho-H3 antibody and propidium iodide. All phases of the cell cycle and the cell death- associated sub-G1 phase were indicated. Data of one representative experiment is shown (Average data can be found in Fig. 6): After 96h the majority of treated BON cells (70.2% versus 0.71% in DMSO control) has accumulated in Sub-G1 population and mitoses have reduced to 0.1% (versus 1.26% in DMSO control) of counted cells (A). In treated KRJ-1 cell culture, the sub-G1 population has tripled (26.6% versus 9.24% of DMSO control). Cells in mitosis phase of the cell cycle have dwindled to 0.084% within the siomycin A treated cells compared to 0.45% in the DMSO group after 96h (B). QGP-1 cells showed a 10-fold increase (18.0% versus 1.77% in DMSO control) of the sub-G1 population after 96h, and a decrease of mitotic cells from 1.08% in DMSO to 0.078 in the siomycin A treated population (C). Following 96h of treatment, in LCC-18 cells mitoses have diminished from 1.12% in the DMSO control to 0.085% under siomycin A and the sub-G1 population has increased 6-fold to 55.3% (versus 9.21% in DMSO control; D).

**Tissue type**		primary	metastasis	total
primary	ileum	44	7	51
	colon	19	0	19
	rectum	11	0	11
	pancreas	41	2	43
	other	7	0	7
total		122	9	131

**Grading**		**G1**	**G2**	**G3**	**total**
primary	Ileum	38	12	1	51
	Colon	9	2	8	19
	Rectum	4	1	6	11
	Pancreas	30	10	3	43
	other	4	2	1	7
primary	pancreatic	30	10	3	43
	gastrointestinal	55	17	16	88
differentiation	well	85	27	0	112
	poorly	0	0	19	19
metastatic status	M0	65	11	5	81
	M1	17	16	14	47
	N/A	3	0	0	3
total		85	27	19	131

**differentiation**		well-differentiated	poorly-differentiated	total
primary	Ileum	50	1	51
	Colon	11	8	19
	Rectum	5	6	11
	Pancreas	40	3	43
	other	6	1	7

**Sex**	male	female	total
	73	58	131

**Age**	Age: <=50	Age: 51-60	Age: 61-70	Age: >=71	total
	34	43	32	22	131

Suppl. 3: Detailed clinicopathological data of immunohistochemically analyzed gastrointestinal and pancreatic GEP-NENs: Distribution of localization, grading, differentiation, primary tumors and metastases; age and sex of the patients

Localization of the primary		pancreatic	ileal	unknown	Total
tissue	primary	4	4	0	8
	metastasis	4	13	1	18
Total		8	17	1	26

Localization of metastases		lymph node MTS	distant MTS	Total
tissue	metastasis	8	10	18

Pre-treatment	frequency	percent
no pre-treatment or surgery	6	40
chemotherapy	1	6,7
PRRT	8	53,3
Total	15	100,0

Age	Age: <=50	Age: 51-60	Age: 61-70	Age: >=71	total
	1	5	5	4	15

sex	male	female	total
	6	9	15

Suppl. 4: Clinicopathological data of primary (fresh frozen) GEP-NEN tissues, analyzed by western blot: Distribution of localization, origin of metastases; clinical data of the patients: sex, age (time point of biopsy) and pre-treatment
